# Rectal artery embolization: recent advances and research progress

**DOI:** 10.3389/fmed.2026.1921217

**Published:** 2026-08-18

**Authors:** Mu Ding, Yongzhen Zhang, Xinqiang Han, Xuemin Wang

**Affiliations:** 1 Department of Interventional Medicine and Vascular Surgery, Shandong Medical and Pharmaceutical University Hospital, Binzhou, Shandong, China; 2 Department of Gastroenterology, Shandong Medical and Pharmaceutical University Hospital, Binzhou, Shandong, China

**Keywords:** embolic materials, hemorrhoidal artery embolization, hemorrhoids, rectal artery embolization, superior rectal artery

## Abstract

Rectal artery embolization (RAE), or the Emborrhoid technique, is a new minimally invasive treatment for bleeding internal hemorrhoids. This review examines vessel selection, embolic materials, procedural success, recurrence, and safety. Technical success involves successful catheterization and occlusion of target arteries, while clinical success means symptom improvement without further treatment. Studies show consistently high technical success but variable clinical success. One meta-analysis found mean technical and clinical success rates of 97.8% and 78.9%, respectively, while another reported pooled estimates of 99% and 82%. Definitions, sample sizes, and follow-up vary across studies. In an 80-patient study, 68.7% had no rectal bleeding after 12 months, while 31.3% experienced recurrence, and 21.3% needed repeat embolization. A separate study with 42 patients showed 93% success but a 54% rate of minor mucosal injury. Current evidence favors superselective superior rectal artery embolization as the primary approach, with selective assessment of middle rectal artery inflow. There's insufficient evidence to broadly recommend large microspheres and liquid agents. Standardized outcomes, clear denominators, and longer follow-up are needed.

## 1 Introduction

Hemorrhoidal disease is among the most common benign anorectal disorders in humans, characterized by engorgement, venous stasis, and enlargement of venous plexuses, with prevalence rates reported to be as high as 40% in the general population (1). Notably, approximately 40% of affected individuals remain asymptomatic, while recurrent rectal bleeding serves as the primary symptom that prompts individuals to seek medical attention and consider advanced treatment options. Standard therapeutic interventions encompass dietary modifications, pharmacological treatments with venoactive agents, rubber band ligation, sclerotherapy, infrared coagulation, Doppler-guided hemorrhoidal artery ligation, stapled hemorrhoidopexy, and hemorrhoidectomy. Clinical guidelines typically advocate for conservative management as the initial therapeutic approach (2, 3). Nonetheless, approximately 10% of patients eventually require surgical intervention. While surgical hemorrhoidectomy is highly efficacious, it is often accompanied by postoperative pain, urinary retention, wound-related complications, delayed recovery, and a diminished willingness among patients to undergo the procedure. Furthermore, the postoperative care associated with this surgery can be relatively burdensome. These limitations have, to some degree, spurred the advancement of novel minimally invasive therapeutic approaches (4).

The endovascular concept was initially introduced by Vidal in 2014. RAE selectively occludes arterial inflow to the hemorrhoidal plexus to control bleeding and improve associated symptoms. Because it avoids direct trauma to the anal cushions, anal canal, and sphincter complex, it may be considered for patients with bleeding-predominant disease that persists despite conservative treatment, particularly when conventional surgery is contraindicated or declined (5–7).

Patient selection should follow proctologic evaluation confirming hemorrhoids as the source of chronic bleeding and excluding alternative causes, including anorectal neoplasia, inflammatory bowel disease or proctitis, and rectal varices. The best-supported candidates are patients with bleeding-predominant grade II-III internal hemorrhoids refractory to conservative or office-based treatment, especially those who decline surgery or have high operative risk, long-term antiplatelet or anticoagulant therapy, or a coagulation disorder; grade IV disease may be considered only when surgery is contraindicated because embolization does not correct fixed prolapse (5, 7).

Major contraindications include anorectal cancer and the usual contraindications to angiography, such as severe renal failure, iodinated-contrast allergy. External or acutely thrombosed hemorrhoids, active anorectal infection or inflammation, and advanced atherosclerosis are additional poor-selection or relative contraindication factors (5, 7).

The initial clinical applications predominantly concentrated on the embolization of the terminal branches of the superior rectal artery (SRA). Subsequently, a variety of embolization strategies have been explored. According to the current body of evidence, RAE has advanced from the phase of assessing technical feasibility to the validation of its perioperative safety and mid-term clinical efficacy (8–10). Nonetheless, several clinically pertinent issues remain unresolved: determining the optimal embolic agent; assessing whether smaller or larger particles offer a superior balance between hemostatic efficacy and safety; deciding whether embolization should be restricted solely to the SRA; and addressing the management of collateral circulation from the middle rectal artery (MRA).

## 2 Narrative review methods

This narrative review was based on a focused PubMed search updated through July 20, 2026, using terms related to hemorrhoids and embolization techniques. Reference lists from relevant articles and reviews were also examined. The review included English-language human studies, systematic reviews, meta-analyses, and pertinent anatomical or technical reports, prioritizing studies with detailed patient data, target vessels, embolic materials, outcomes, follow-up, or adverse events. The comparative characteristics of the embolic materials considered in this review are summarized in Table 1. Studies not related to hemorrhoidal embolization or lacking relevant data were excluded. Studies not related to hemorrhoidal embolization or lacking relevant data were excluded. No new meta-analysis or formal risk-of-bias assessment was conducted, as the goal was interpretive synthesis. Evidence was descriptively synthesized. Technical success meant achieving catheterization and occlusion of target branches per each study's angiographic endpoint. Clinical success was defined by the absence or improvement of bleeding or hemorrhoid symptoms without further treatment, according to the source study. Denominators, follow-up duration, and study design are noted when available. Core studies are summarized in Table 2.

**Table 1:** Comparative characteristics of embolic materials used in rectal artery embolization.

Embolic material	Common specifications/ particle size	Procedural controllability	Resorbability	Typical tissue-level effect	Main advantages	Main limitations	Current interpretation
Microcoils (pushable or detachable coils)	Commonly 0.018-inch system; 2–3 mm coils	High	No	Predominantly proximal or branch-level occlusion; relatively low risk of deep mucosal ischemia	Precise deployment, clear angiographic localization, controllable release, and relatively wide safety margin	May leave residual distal arterial flow or collateral supply untreated; recurrence is often related to distal reperfusion or collateral inflow	Most suitable when procedural safety is prioritized
Gelatin sponge plus coils	Commonly 350–560 μm	Moderate	Yes	Relatively distal but temporary and potentially milder embolization	Theoretically lower ischemic risk; low cost; useful as an adjunct to coils	Long-term risk of recanalization or reperfusion remains uncertain	A reasonable combined strategy, but long-term superiority has not been established
TAGM microspheres plus coils	Commonly 300–500 μm	Moderate to high	No	More homogeneous distal embolization	Calibrated particle size and relatively predictable distal penetration	Excessively distal embolization may increase mucosal injury	Potential option only with cautious procedural endpoints; evidence for a preferred size is limited to a single-center 42-patient trial
TAGM microspheres alone	500–700 μm, 700–900 μm, or 900–1200 μm	Moderate to high	No	Predominantly distal terminal-arterial embolization; smaller particles penetrate more deeply	Maximizes distal devascularization and may provide stronger hemodynamic suppression	Higher rates of ulceration or fibrotic mucosal changes have been reported; safety window may be narrower than coil-based strategies	Should not be broadly adopted outside strict procedural endpoints and careful endoscopic/clinical monitoring
Liquid embolic agents/adhesive embolics such as EVOH or NBCA	No mature standardized protocol in hemorrhoidal disease	Moderate to low	No	Potentially deep, permanent, and irreversible ischemic occlusion	Theoretically complete vascular obliteration	EVOH caused distal rectal necrosis in 3/3 animals in a porcine study; human evidence for EVOH and NBCA is sparse	Not recommended for routine use; EVOH and NBCA require separate evaluation and should not be considered interchangeable
Detachable metallic microcoils including bare platinum or electrolytically detachable coils	Bare platinum or electrolytically detachable coil systems	Very high	No	Similar to conventional coils, but with more controlled deployment	Improved precision, repositioning capability, and procedural control	Higher cost and limited availability	Particularly useful in complex vascular anatomy or when precise distal branch embolization is required

TAGM, tris-acryl gelatin microspheres; EVOH, ethylene-vinyl alcohol copolymer; NBCA, N-butyl cyanoacrylate.

**Table 2:** Core studies informing this narrative review.

Study	Design and sample	Target/material	Outcome and follow-up	Key findings	Principal limitation
De Gregorio et al. (11)	Prospective multicenter registry; *n* = 80	SRA; microcoils, with microspheres for recurrence	Absence/recurrence of bleeding; 12 months	Technical success 100%; no bleeding 55/80 (68.7%); recurrence 25/80 (31.3%); repeat embolization 17/80 (21.3%)	No comparator; retreatment strategy changed the embolic material
Han et al. (12)	Retrospective cohort; *n* = 32	SRA; 350–560 μm gelatin sponge plus 2–3 mm coils	Bleeding resolution and complications; ≥12 months	Technical success 100%; initial bleeding resolution 27/32 (84.4%); rebleeding in 4/27 initial responders (14.8%)	Single-arm study; recurrence denominator differs from total cohort
Pavidapha et al. (14)	Retrospective angiographic research letter; 242 procedures	Descriptive SRA/MRA anatomy	Arterial supply patterns and repeat-intervention anatomy	Isolated bilateral SRA supply 47%; MRA involvement 53%; dominant MRA was frequent at repeat intervention	Short research letter; descriptive, non-comparative, hypothesis-generating evidence
de Oliveira et al. (15)	Prospective single-center pilot; *n* = 10	SRA in 100%; MRA in 80%; microspheres plus microcoils	HSS/QoL and recurrence; 12 months	Technical success 100%; HSS and QoL improved by 88%; no recurrence; one rectal perforation requiring Hartmann procedure	Very small pilot; major complication limits routine MRA extension
De Gregorio et al. (17)	Prospective device-specific cohort; *n* = 21	SRA; electrically detachable bare-platinum microcoils	Clinical improvement and retreatment; 12 months	Technical success 100%; improvement 17/21 (80.9%); repeat embolization 3/21; surgery 1/21	Small single-center study from the same research group as the later registry
Wang et al. (19)	Retrospective multicenter cohort; *n* = 41 (23 vs. 18)	Coils plus gelatin sponge vs coils plus 300–500 μm microparticles	Absence of hematochezia; 6–15 months	Technical success 100%; initial clinical efficacy 20/23 (87.0%) vs. 16/18 (88.9%), *p* = 0.098	Nonrandomized and underpowered for equivalence or superiority
Küçükay and Küçükay (21)	Prospective randomized single-center study; *n* = 42; 14 per arm; 15 grade IV patients overall	SRA; tris-acryl gelatin microspheres 500–700, 700–900, or 900–1200 μm	Clinical success at 12 months; endoscopy and complications	Overall clinical success 93%; minor mucosal injury 23/42 (54%); greatest bleeding-score decrease in 900–1200 μm arm	Only 14 patients per arm; findings should not be generalized to other particles
Vidal et al. (25)	Prospective first clinical series; *n* = 14	SRA; pushable microcoils	Bleeding response and retreatment; early follow-up	Four patients rebled; two were successfully re-embolized	Small heterogeneous high-risk cohort; no comparator
Bagla et al. (28)	Retrospective outpatient cohort; *n* = 134	SRA/MRA as indicated; particles plus microcoils	Clinical success at 1 month	Technical success 133/134 (99%); clinical success 124/134 (93%); 10 repeat embolizations	Very short follow-up and no control group
Falsarella et al. (29)	Randomized clinical trial; *n* = 33 (RAE 16, CFH 17)	SRA embolization vs. closed Ferguson hemorrhoidectomy	Symptoms and pain; 12 months	Similar symptom frequency at 12 months; less perioperative and first-defecation pain after RAE	Small sample; not designed to resolve embolic-material questions

CFH, closed Ferguson hemorrhoidectomy; HSS, Hemorrhoidal Severity Score; IRA, inferior rectal artery; MRA, middle rectal artery; QoL, quality of life; RAE, rectal artery embolization; SRA, superior rectal artery.

## 3 Embolization target: SRA only, or should the MRA also be considered?

The theoretical foundation for RAE is grounded in the vascular hyperflow of hemorrhoidal disease. Internal hemorrhoids are characterized by the enlargement of vascular cushions, increased arterial inflow, venous stasis, and mucosal redundancy. The SRA is generally considered the primary arterial supply to the internal hemorrhoidal plexus. Selective embolization of its terminal branches can diminish arterial inflow, alleviate hemorrhoidal venous congestion, and ameliorate bleeding symptoms (5). Most studies to date have identified the SRA as the primary, or even sole, target vessel for RAE. In the study conducted by Gregorio, embolization of the SRA was initially performed using microcoils in all 80 cases. Although this approach resulted in the complete resolution of rectal bleeding in 68.7% of patients at 12 months, and 21.3% of patients who experienced recurrent bleeding required repeat embolization with additional microspheres (11). According to this evidence, while an embolization strategy aimed at the SRA is possible, it may not adequately address all arterial sources supplying the hemorrhoidal region. In our earlier research, a major reason for treatment failure was found to be the failure to identify and address collateral arterial supply from the MRA or inferior rectal artery (IRA) during the initial procedure (12). In our study, 4 patients (14.8%) experienced recurrent bleeding within 6 months. Subsequent bilateral internal iliac angiography confirmed collateral arterial supply to the hemorrhoidal region from the internal pudendal artery and its downstream branches. Following additional embolization of these branches, no further bleeding was observed. Panneau also proposed that ongoing perfusion from a dominant MRA may be a critical factor in treatment failure (13). Pavidapha et al. (14) reported a retrospective angiographic analysis of 242 procedures in a short research letter: isolated bilateral SRA supply was present in 47%, whereas 53% showed MRA involvement. Among 21 patients undergoing repeat intervention, 17 had MRA inflow during the initial angiogram, and 19 had a dominant MRA at repeat embolization. These descriptive findings generate hypotheses but don't prove that routine MRA embolization improves outcomes.

**Figure 1:**
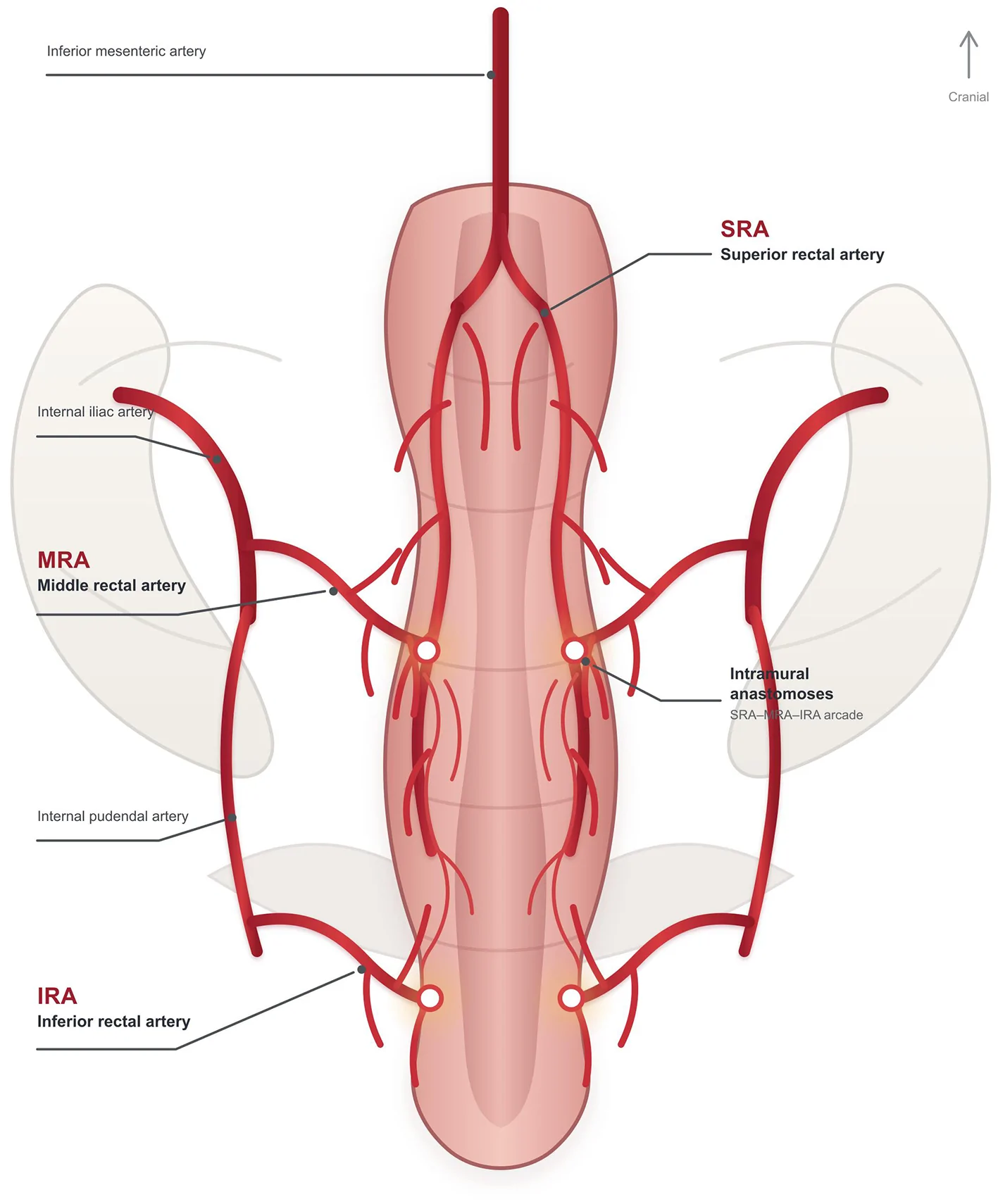
Schematic illustration of the superior (SRA), middle (MRA), and inferior rectal arteries (IRA) and their principal intramural anastomoses.

Consequently, although the SRA remains the primary target vessel in the Emborrhoid procedure, the strategy should not be confined to the simple occlusion of the SRA trunk. Instead, it should prioritize a systematic assessment of the arterial supply anatomy and ensure adequate embolization of the responsible vessels. Specifically, when intraoperative angiography indicates asymmetric or absent perfusion of the hemorrhoidal cushion, or when the MRA is suspected to be comparable to or larger than the SRA in caliber, further angiography of the internal iliac artery should be conducted to evaluate MRA inflow. The SRA, MRA, and IRA relationships are summarized schematically in Figure 1.

However, this does not suggest that routine additional MRA embolization is justified in all cases. Oliveira conducted a preliminary prospective study on the combined use of SRA and MRA embolization, wherein MRA embolization was performed in 80% of the 10 patients involved. The study protocol used 1,100-μm Embozene microspheres to near stasis followed by 3-mm × 15-cm POD microcoils in both the SRA and contributing MRA branches. No clinical recurrence was observed within 12 months, and both the Hemorrhoidal Severity Score and quality-of-life metrics showed significant improvement. Nonetheless, one patient, contrast extravasation occurred during a wedged manual injection into the left MRA. Although hemostasis was achieved with additional coil embolization, the event ultimately resulted in ischemic rectal perforation and sepsis 17 days after the procedure, necessitating Hartmann's procedure (15). This safety concern indicates that expanding the target vascular territory may reduce the therapeutic safety margin and result in severe complications.

Consequently, the selection of target vessels should not be simplified to a binary decision between “SRA only” and “a combination of SRA and MRA.” Instead, superselective and complete embolization of the SRA should remain the primary strategy. In patients presenting with angiographic evidence of a hypertrophic MRA, prominent anastomotic channels, competitive arterial flow, or early recurrence after initial embolization, the MRA should be specifically evaluated and selectively treated when appropriate (13, 16).

## 4 Embolic materials and particle size

Currently, embolic materials for RAE include coils, Gelatin sponge, and microspheres. The selection aims to ensure adequate distal devascularization of the hemorrhoidal plexus while preventing mucosal damage from excessive ischemia.

### 4.1 Microcoils

Coils were the first embolic agents used in RAE studies and are still commonly used today (6, 11, 17). Coil embolization is simple and allows precise blockage of targeted arteries, minimizing the risk of excessive penetration into the rectal mucosa. A study conducted by Gregorio on detachable platinum microcoils showed 100% technical success, with 80.9% of patients improving and no major complications (17).

However, coils may cause proximal or segmental occlusion, potentially missing distal anastomoses, leading to recurrent bleeding if distal flow or collateral channels remain untreated. Evidence shows that technical success with coils does not always prevent recurrence and may not lead to permanent clinical success (11, 12).

### 4.2 Gelatin sponge particles and microspheres

Gelatin sponge is an absorbable material used alone or with coils to produce temporary distal flow reduction; its resorption may reduce the duration of ischemia but can permit recanalization. Calibrated microspheres produce more durable distal devascularization but may increase mucosal ischemia or ulceration when particles are small, injection is excessively distal, or the embolization endpoint is aggressive (8, 18, 19). In previous work, we evaluated protocols using coils (2–3 mm) plus 350–560 μm gelatin sponge particles (*n* = 23) were compared with coils plus 300–500 μm microparticles (*n* = 18). Technical success was 100% in both groups; initial clinical efficacy was 20/23 (87.0%) and 16/18 (88.9%), respectively (*p* = 0.098) (19). Because allocation was not randomized and the sample was small, these data do not establish equivalence or superiority of either particle type.

### 4.3 Combination embolization: particles and coils

Combined embolization uses particles for distal flow reduction and coils for controlled branch occlusion. In the Makris meta-analysis of 14 studies (*n* = 362), mean technical and clinical success were 97.8% (SD, 3.5) and 78.9% (SD, 10.5), respectively. Study-level subgroup averages for rebleeding were 21.5% with coils alone (*n* = 111; SD, 18.2; range, 0%−44%) and 10.05% with coils plus particles (*n* = 108; SD, 4.8; range, 5%−15.7%; *p* < 0.0001) (8). Because the contributing studies were heterogeneous and predominantly observational, this subgroup comparison is hypothesis-generating and should not be interpreted as randomized evidence of comparative superiority. Particles may be considered when angiography shows abundant terminal branches or persistent distal staining (1, 5).

A 2024 transanal echo-Doppler study found that distal embolization more effectively reduced postoperative hemorrhoidal arterial flow velocity compared to proximal embolization. However, this did not lead to significantly better short-term symptom relief, suggesting that the link between reduced blood flow and clinical benefit may not be straightforward (20).

### 4.4 Particle size: smaller is not always better

Particle size is a key debate in RAE research, with greater biological significance than the type of embolic agent. A study conducted by Küçükay divided triacryl gelatin microspheres into three sizes: 500–700 μm, 700–900 μm, and 900–1200 μm. Smaller particles improved bleeding control faster but increased postoperative pain and mild ischemic injury. Conversely, 900–1200 μm particles acted slower but provided the best bleeding control at 12 months, with minimal postoperative pain and ischemic changes (21). The study showed a 93% overall clinical success rate, with the most significant 12-month reduction in bleeding scores seen in the 900–1200 μm group. However, 54% of patients experienced minor mucosal issues like superficial ulcers or small scars. With just 14 patients per particle-size group, the study suggests further research on larger microspheres but doesn't determine an optimal size. The results are specific to tris-acryl gelatin microspheres and shouldn't be generalized to other particles with different properties. Thus, it should be tailored to the embolization goal and the observed vascular anatomy.

### 4.5 Liquid embolic agents and detachable coil systems

Ethylene-vinyl alcohol copolymer (EVOH) and N-butyl cyanoacrylate (NBCA) are permanent liquid embolic agents, but their use in hemorrhoidal disease is not well-supported and they are not interchangeable. In a study with nine pigs, EVOH caused rectal necrosis, unlike microspheres and microcoils (18). This suggests EVOH should not be routinely used. NBCA lacks strong clinical evidence and standardized protocols for hemorrhoidal treatment.

Detachable bare-platinum microcoils allow for controlled release and repositioning before detachment. A single-center study with 21 patients showed 100% technical success, 80.9% clinical improvement, 14.3% required repeat embolization, and 4.8% needed surgery after 12 months (17). This study, separate from an 80-patient Spanish registry by the same research group, confirms procedural feasibility but not superiority over pushable coils (11).

## 5 Hemostatic efficacy and overall clinical outcomes

In 2015, Vidal et al. presented a study on 14 patients, showing that RAE is technically feasible, safe, and tolerable. Later research indicated promising early results in controlling bleeding, highlighting its potential in treating chronic hemorrhoidal bleeding and related symptoms.(6, 22–25). Studies indicate high technical success and significant bleeding reduction, though clinical success varies with definitions and follow-up duration (11, 12, 26, 27). Nguyenhuy et al. (9) pooled 13 studies (381 patients) and reported technical success of 99% (95% CI, 94%-100%) and clinical efficacy of 82% (95% CI, 73%-89%). Makris et al. (8) summarized 14 studies (362 patients) and reported mean technical success of 97.8% (SD, 3.5) and mean clinical success of 78.9% (SD, 10.5) (9). These estimates should be interpreted in the context of heterogeneous definitions, embolization protocols, and follow-up intervals.

The prospective Spanish Emborrhoid Registry enrolled 80 patients with Goligher grade I-III bleeding. Technical success, defined as angiographic occlusion of all distal SRA branches, was achieved in all patients. Clinical effectiveness was evaluated at 1, 3, 6, and 12 months using clinical examination, anoscopy, bleeding recurrence, a visual analog score, quality-of-life assessment, patient satisfaction, and the need for repeat embolization or surgery. At 12 months, 55/80 (68.7%) had no rectal bleeding, 25/80 (31.3%) had recurrent bleeding, 17/80 (21.3%) underwent repeat embolization, and 4/80 (5%) underwent open hemorrhoidectomy (11). In an independent retrospective outpatient cohort of 134 patients, efficacy was assessed by comparing baseline and 1-month hemorrhoid-related pain, Hemorrhoidal Severity Score, quality-of-life score, French Bleeding Score, and Goligher grade. Clinical success was defined as symptomatic improvement without additional treatment and was achieved in 124/134 patients (93%), whereas 10 patients required repeat embolization (28). These findings represent short-term, uncontrolled evidence.

A 2023 randomized trial of 33 patients compared RAE (*n* = 16) with Closed Ferguson hemorrhoidectomy (CFH; *n* = 17). Symptom frequencies did not differ significantly at 12 months. However, the RAE group had less perioperative pain, lower pain at first defecation, and lower analgesic use (29). The small sample supports a potential pain advantage but is insufficient to establish broad comparative equivalence.

To date, no completed head-to-head study has reported comparative outcomes between RAE and Doppler-guided hemorrhoidal artery ligation.

## 6 Follow-up and recurrence pattern

Follow-up protocols differ among studies but generally involve clinical assessments at 1, 3, 6, and 12 months post-procedure (11, 15, 19, 26, 27). Common outcome measures include bleeding and hemorrhoidal severity scores, Goligher grade, quality of life, patient satisfaction, transfusion needs, and requirements for repeat embolization or additional surgery (11, 20, 21, 30).

Recurrence after RAE is common, often defined by renewed rectal bleeding, inadequate bleeding score improvement, or the need for further treatment (10–12, 28). Early recurrence (within 6 months) may suggest incomplete embolization or untreated collateral supply, while recurrence at 1 year may indicate new collateral circulation or disease progression (11, 20). Published recurrence and retreatment estimates vary with definition and follow-up; the detailed 12-month Spanish registry results are reported in Section 5 rather than repeated here. Technical success should therefore be separated from durable bleeding control, recurrence, and freedom from retreatment.

A key limitation in current research is the scarcity of high-quality data beyond 12 months, with a lack of follow-up studies extending to 24 months or more. Future research should focus on long-term outcomes to better assess the durability of RAE.

## 7 Complications and safety

### 7.1 General safety profile

Most studies report a good safety profile, with typically mild and self-limiting adverse events such as anal discomfort, pelvic pain, tenesmus, low-grade fever, nausea, vomiting, access-site hematoma, and temporary post-procedural pain (11, 12, 19, 27, 29, 31). Rarely, severe complications like rectal or rectosigmoid ischemia can occur (15, 32).

The safety of RAE depends on the extent of embolization and whether it includes the MRA and/or IRA. Küçükay found that smaller particles can cause mild ischemic changes, like superficial ulceration and scarring (21). Oliveira reported that combined distal SRA and MRA embolization led to rectal ischemia and perforation in 1 of 10 patients, highlighting increased risks with more extensive embolization (15). Although severe complications are rare, they are more likely with broader and more distal embolization strategies.

### 7.2 Anal discomfort, pain, fever, and tenesmus

Anal discomfort and tenesmus are clinically relevant because RAE aims to reduce post-procedural pain compared to excisional surgery. Our earlier 32-patient study reported transient mild-to-moderate pain in 4/32 (12.5%), low-grade fever in 11/32 (34.4%), and tenesmus in 17/32 (53.1%); all resolved without further treatment (12). These findings indicate that transient anorectal irritation is common after RAE.

The randomized trial by Falsarella suggests that SRA embolization can lessen post-procedural pain and the need for painkillers compared to closed Ferguson hemorrhoidectomy (29). Thus, Emborrhoid may be most beneficial for patients with bleeding-dominant hemorrhoids who want to avoid the pain of excisional surgery.

### 7.3 Rectal mucosal bleeding

Rectal mucosal bleeding following RAE is a significant but not fully standardized safety concern. In RAE studies, post-procedure bleeding is often noted as recurrent hematochezia or hemorrhoidal bleeding, while endoscopically confirmed mucosal bleeding from ischemic ulcers is rarely identified as a distinct issue (11, 12, 28). It's important to differentiate between recurrent hemorrhoidal bleeding, indicating incomplete hemostasis or collateral blood supply, and ischemic mucosal injury, a genuine procedure-related complication.

In the 42-patient randomized particle-size study, 23/42 (54%) developed minor mucosal findings: 19/42(45%) had small superficial ulcers, 3/42(7%) had small rectosigmoid-junction ulcers, and 1/42 (2%) had a small fibrotic scar (21). These results indicate that minor endoscopic mucosal injuries may occur more frequently than clinically noticeable ischemia, especially with the use of particulate embolic agents.

## 8 Future directions

The field needs larger, standardized prospective studies. Future research should focus on identifying the optimal embolic agent, determining the safest and most effective particle size, assessing the role of combined particle-plus-coil embolization, evaluating the benefits of routine vs. selective MRA, and finding the best approach for managing recurrent bleeding (1, 7, 8, 13, 33).

Future trials should standardize various factors such as baseline disease grading, bleeding and hemorrhoid severity scores, quality-of-life assessments, embolic materials, particle size, number of embolized branches, use of cone-beam CT, MRA angiography, recurrence definitions, and adverse event classifications (10, 16, 20, 30). Additionally, long-term follow-up beyond 12 months is necessary to assess durability and compare RAE with other treatments like rubber band ligation, Doppler-guided hemorrhoidal artery ligation, stapled hemorrhoidopexy, and excisional hemorrhoidectomy (10, 13, 29, 30, 33).

## 9 Conclusions

RAE is a minimally invasive option for bleeding-predominant internal hemorrhoids, with consistently high technical success but more variable clinical durability. Recurrence may reflect incomplete distal treatment, collateral MRA or IRA inflow, or disease progression; current evidence is dominated by heterogeneous single-arm cohorts.

When selecting embolic agents, it's important to consider controllability, distal devascularization, durability, and mucosal safety. Coils allow precise deployment but may not prevent distal or collateral flow, while particles can reach more distal vessels but risk mucosal injury. The supposed benefit of 900–1200 μm tris-acryl gelatin microspheres is based on a single trial with 42 patients, which is insufficient for broad recommendations. Routine use of liquid agents is not advised: EVOH caused distal rectal necrosis in all three animals in a nine-pig study, and clinical evidence for NBCA is limited (18).

Superselective SRA embolization should remain the primary approach. MRA angiography and embolization should be individualized and reserved for demonstrable collateral or dominant supply (16, 34). Multicenter comparative studies with standardized definitions, explicit denominators, and follow-up beyond 12 months are needed before any embolic material, particle size, or routine MRA strategy can be preferred.

## References

[1] Talaie R Torkian P Moghadam AD Tradi F Vidal V Sapoval M . Hemorrhoid embolization: a review of current evidences. Diagn Interv Imaging. (2022) 103:3–11. doi: 10.1016/j.diii.2021.07.001 34456172

[2] Hawkins AT Davis BR Bhama AR Fang SH Dawes AJ Feingold DL . The American Society of Colon and Rectal Surgeons clinical practice guidelines for the management of hemorrhoids. Dis Colon Rectum. (2024) 67:614–23. doi: 10.1097/DCR.0000000000003276 38294832

[3] Tarasconi A Perrone G Davies J Coimbra R Moore E Azzaroli F . Anorectal emergencies: WSES-AAST guidelines. World J Emerg Surg. (2021) 16:48. doi: 10.1186/s13017-021-00384-x 34530908 PMC8447593

[4] Sandler RS Peery AF. Rethinking what we know about hemorrhoids. Clin Gastroenterol Hepatol. (2019) 17:8–15. doi: 10.1016/j.cgh.2018.03.020 29601902 PMC7075634

[5] Panneau J Mege D Di Bisceglie M Duclos J Habert P Bartoli A . Rectal artery embolization for hemorrhoidal disease: anatomy, evaluation, and treatment techniques. RadioGraphics. (2022) 42:1829–44. doi: 10.1148/rg.220014 36190848

[6] Vidal V Louis G Bartoli JM Sielezneff I. Embolization of the hemorrhoidal arteries (the Emborrhoid technique): a new concept and challenge for interventional radiology. Diagn Interv Imaging. (2014) 95:307–15. doi: 10.1016/j.diii.2014.01.016 24589187

[7] Zakavi SS Mirza-Aghazadeh-Attari M Mansur A Habibollahi P Nezami N Camacho JC. Rectal artery embolization for the treatment of hemorrhoidal disease. Semin Intervent Radiol. (2025) 42:93–100. doi: 10.1055/s-0044-1801360 40342391 PMC12058288

[8] Makris GC Thulasidasan N Malietzis G Kontovounisios C Saibudeen A Uberoi R . Catheter-directed hemorrhoidal dearterialization technique for the management of hemorrhoids: a meta-analysis of the clinical evidence. J Vasc Interv Radiol. (2021) 32:1119–27. doi: 10.1016/j.jvir.2021.03.548 33971251

[9] Nguyenhuy M Xu Y Kok HK Maingard J Joglekar S Jhamb A . Clinical outcomes following rectal artery embolisation for the treatment of internal haemorrhoids: a systematic review and meta-analysis. Cardiovasc Intervent Radiol. (2022) 45:1351–61. doi: 10.1007/s00270-022-03154-7 35551442

[10] Al Jnainati M Ikkurthy N Ayoub M Shahid A Baig MT Iltaf M . Outcomes of embolization therapy of superior rectal arteries for the management of grade 1 to 3 internal hemorrhoids: a systematic review of clinical studies. Int J Colorectal Dis. (2025) 40:190. doi: 10.1007/s00384-025-04944-4 40877526 PMC12394367

[11] De Gregorio MA Guirola JA Serrano-Casorran C Urbano J Gutiérrez C Gregorio A . Catheter-directed hemorrhoidal embolization for rectal bleeding due to hemorrhoids (Goligher grade I-III): prospective outcomes from a Spanish Emborrhoid registry. Eur Radiol. (2023) 33:8754–63. doi: 10.1007/s00330-023-09923-3 37458757

[12] Han X Xia F Chen G Sheng Y Wang W Wang Z . Superior rectal artery embolization for bleeding internal hemorrhoids. Tech Coloproctol. (2021) 25:75–80. doi: 10.1007/s10151-020-02312-8 32712932

[13] Panneau J Mege D Di Bisceglie M Duclos J Khati I Vidal V . Hemorrhoidal disease: what role can rectal artery embolization play? Front Surg. (2025) 11:1474799. doi: 10.3389/fsurg.2024.1474799 39840267 PMC11747564

[14] Pavidapha A Sajan A Lerner J Bagla S Kasimcan MO Isaacson A . Angiographic analysis of anatomical variants in 242 hemorrhoidal artery embolization. J Vasc Interv Radiol. (2025) 36:913–5. doi: 10.1016/j.jvir.2025.01.003 39814171

[15] de Oliveira DS Hora JAB de Assis AM Moreira AM Nahas SC Carnevale FC. Embolization of rectal arteries for treating hemorrhoidal disease using a combination of microspheres and microcoils: a pilot study. Cardiovasc Intervent Radiol. (2025) 48:388–94. doi: 10.1007/s00270-024-03942-3 39725725

[16] Lindquist J Hart J Marchak K Bent Robinson E Trivedi P. Imaging for hemorrhoidal disease: navigating rectal artery embolization from planning to follow-up. Semin Intervent Radiol. (2024) 41:263–9. doi: 10.1055/s-0044-1788056 39165649 PMC11333117

[17] De Gregorio MA Bernal R Ciampi-Dopazo JJ Urbano J Millera A Guirola JA. Safety and effectiveness of a new electrical detachable microcoil for embolization of hemorrhoidal disease, November 2020–December 2021: results of a prospective study. J Clin Med. (2022) 11:3049. doi: 10.3390/jcm11113049 35683436 PMC9181639

[18] Tradi F Panneau J Brige P Mege D Habert P Hak JF . Evaluation of multiple embolic agents for embolization of the superior rectal artery in an animal model. Cardiovasc Intervent Radiol. (2022) 45:510–9. doi: 10.1007/s00270-021-03041-7 34988702

[19] Wang X Sheng Y Wang Z Wang W Xia F Zhao M . Comparison of different embolic particles for superior rectal arterial embolization of chronic hemorrhoidal bleeding: Gelfoam versus microparticle. BMC Gastroenterol. (2021) 21:465. doi: 10.1186/s12876-021-02046-3 34906095 PMC8670118

[20] Tutino R Stecca T Farneti F Massani M Santoro GA. Transanal eco-Doppler evaluation after hemorrhoidal artery embolization. World J Gastroenterol. (2024) 30:2332–42. doi: 10.3748/wjg.v30.i17.2332 38813050 PMC11130570

[21] Küçükay MB Küçükay F. Superior rectal artery embolization with tris-acryl gelatin microspheres: a randomized comparison of particle size. J Vasc Interv Radiol. (2021) 32:819–25. doi: 10.1016/j.jvir.2021.02.011 33640516

[22] Sun X Xu J Zhang J Jin Y Chen Q. Management of rectal bleeding due to internal haemorrhoids with arterial embolisation: a single-centre experience and protocol. Clin Radiol. (2018) 73:985.e1-985.e6. doi: 10.1016/j.crad.2018.07.105 30149946

[23] Tradi F Louis G Giorgi R Mege D Bartoli JM Sielezneff I . Embolization of the superior rectal arteries for hemorrhoidal disease: prospective results in 25 patients. J Vasc Interv Radiol. (2018) 29:884-892.e1. doi: 10.1016/j.jvir.2018.01.778 29724519

[24] Moussa N Sielezneff I Sapoval M Tradi F Del Giudice C Fathallah N . Embolization of the superior rectal arteries for chronic bleeding due to haemorrhoidal disease. Colorectal Dis. (2017) 19:194–9. doi: 10.1111/codi.13430 27338153

[25] Vidal V Sapoval M Sielezneff Y De Parades V Tradi F Louis G . Emborrhoid: a new concept for the treatment of hemorrhoids with arterial embolization: the first 14 cases. Cardiovasc Intervent Radiol. (2015) 38:72–8. doi: 10.1007/s00270-014-1017-8 25366092

[26] Jiang T Fan L Tang X Xu Z Wu W. Superselective superior rectal artery embolization in the treatment of hemorrhoidal disease. Front Med. (2025) 12:1530981. doi: 10.3389/fmed.2025.1530981 PMC11897518 40078395

[27] Stecca T Farneti F Balestriero G Barban M Caratozzolo E Zilio S . Superior rectal artery embolization for symptomatic grades 2 and 3 hemorrhoidal disease: 6-month follow-up among 43 patients. J Vasc Interv Radiol. (2021) 32:1348–57. doi: 10.1016/j.jvir.2021.06.005 34166805

[28] Bagla S Pavidapha A Lerner J Kasimcan MO Piechowiak R Josovitz K . Outcomes of hemorrhoidal artery embolization from a multidisciplinary outpatient interventional center. J Vasc Interv Radiol. (2023) 34:745–9. doi: 10.1016/j.jvir.2023.01.023 36736822

[29] Falsarella PM Nasser F Affonso BB Galastri FL Motta-Leal-Filho JMD Valle LGM . Embolization of the superior rectal arteries versus closed hemorrhoidectomy (Ferguson technique) in the treatment of hemorrhoidal disease: a randomized clinical trial. J Vasc Interv Radiol. (2023) 34:736–44.e1. doi: 10.1016/j.jvir.2023.01.022 36736690

[30] Morsi S Linares Bolsegui M Kobeissi H Ghozy S Kallmes DF Kelley SR . Common design and data elements on rectal artery embolization for treatment of symptomatic internal hemorrhoidal disease: an interactive systematic review of clinical trials. CVIR Endovasc. (2024) 7:45. doi: 10.1186/s42155-024-00458-2 38733497 PMC11088570

[31] Campennì P Iezzi R Marra AA Posa A Parello A Litta F . The Emborrhoid technique for treatment of bleeding hemorrhoids in patients with high surgical risk. J Clin Med. (2022) 11:5533. doi: 10.3390/jcm11195533 36233395 PMC9571675

[32] Eberspacher C Ficuccilli F Tessieri L D'Andrea V Lauro A Fralleone L . Annoyed with haemorrhoids? Risks of the Emborrhoid technique. Dig Dis Sci. (2021) 66:3725–9. doi: 10.1007/s10620-021-07208-7 34398325

[33] Lei ML Dong LL Zhang HP Yu YB. Does hemorrhoidal artery embolization really benefit patients with hemorrhoids? World J Gastroenterol. (2024) 30:4569–75. doi: 10.3748/wjg.v30.i42.4569 39563747 PMC11572626

[34] Falsarella PM Katz M Affonso BB Galastri FL Arcuri MF Motta-Leal-Filho JMD . Angiographic description of the superior rectal artery and its anatomical variations in patients undergoing embolization of the superior rectal arteries in hemorrhoidal disease treatment. Einstein. (2024) 22:eAO0688. doi: 10.31744/einstein_journal/2024AO0688 39356943 PMC11461003

